# Fatty liver mediates the association of hyperuricemia with prediabetes and diabetes: a weighting-based mediation analysis

**DOI:** 10.3389/fendo.2023.1133515

**Published:** 2023-04-12

**Authors:** Til Bahadur Basnet, Shanshan Du, Ruimei Feng, Jie Gao, Jiamin Gong, Weimin Ye

**Affiliations:** ^1^ Department of Epidemiology and Health Statistics, School of Public Health, Fujian Medical University, Fuzhou, China; ^2^ Department of Medical Epidemiology and Biostatistics, Karolinska Institutet, Stockholm, Sweden

**Keywords:** hyperuricemia (HUA), prediabetes, diabetes, multiple mediators, China

## Abstract

**Background:**

Fatty liver, obesity, and dyslipidemia are associated with prediabetes or diabetes risk, and hyperuricemia co-exists. The present study evaluated the role of multiple mediators, namely, fatty liver, body mass index (BMI), and dyslipidemia, in the association between hyperuricemia and diabetes status.

**Methods:**

Baseline data from the ongoing Fuqing cohort (5,336 participants) were analyzed to investigate the association of hyperuricemia with diabetes status using a multinomial logistic regression model. Furthermore, causal mediation analysis with the weighting-based approach was performed to estimate hyperuricemia’s total natural direct effect (tnde), total natural indirect effect (tnie), and total effect (te) on prediabetes and diabetes risk, mediating jointly *via* fatty liver, BMI, and dyslipidemia.

**Results:**

In multinomial analysis without considering mediators’ effects, hyperuricemia was associated with a higher risk of prediabetes only (odds ratio: 1.25; 95% CI: 1.09–1.43; *p* < 0.001). When fatty liver, BMI, and dyslipidemia were considered as multiple mediators in the association, hyperuricemia was linked to both prediabetes [tnde: 1.11, 95% CI: 1.04–1.11; tnie: 1.07, 95% CI: 1.05–1.09; and overall proportion mediated (pm): 42%, 95% CI: 27%–73%] and diabetes risk (tnde: 0.96, 95% CI: 0.82–1.14; tnie: 1.25, 95% CI: 1.18–1.33; and pm: 100%, 95% CI: 57%–361%). Hyperuricemia showed significant tnde, te, and tnie, mediated by fatty liver jointly with dyslipidemia (pm = 17%) or BMI (pm = 35%), on prediabetes risk.

**Conclusion:**

Hyperuricemia could increase prediabetes or diabetes risk, partially mediated by fatty liver, BMI, and dyslipidemia. Fatty liver is the crucial mediator in the association between hyperuricemia and prediabetes.

## Highlights

- Fatty liver disease singly and combined with body mass index and/or dyslipidemia could mediate the association between hyperuricemia and diabetes. Therefore, fatty liver disease is a crucial mediator in this association.
- The present findings suggest further randomized control trials are needed to consider treatment options for asymptomatic hyperuricemia with higher BMI, dyslipidemia, and fatty liver to prevent prediabetes and diabetes risk.
- Clinicians should be cautious of hyperuricemic patients with higher BMI, dyslipidemia, and fatty liver to avoid the future risk of developing diabetes.

## Introduction

Type 2 diabetes mellitus (T2DM) is a leading public health burden as the incidence and prevalence are substantial worldwide and even increasing. The International Diabetes Federation estimated that the number of T2DM patients worldwide was 463 million in 2019, and age-adjusted prevalence was 8.3% and expected to increase to 9.6% by 2045 among the age group 20–79 years (1). A recent national representative diabetes survey reported that the weighted prevalence of total diabetes, self-reported diabetes, newly diagnosed diabetes, and prediabetes was 12.8% [95% confidence interval (CI) 12.0%–13.6%], 6.0% (5.4%–6.7%), 6.8% (6.1%–7.4%), and 35.2% (33.5%–37.0%), respectively, among adults living in China (2). A varied range of risk factors, such as socioeconomic, dietary, lifestyle, environmental, and genetic factors, are under consideration for prediabetes and diabetes in different populations worldwide (3).

In recent decades, the incidence and prevalence of high serum uric acid (SUA) have increased worldwide. Although high SUA is causally linked to gout, evidence shows that it is also related to several chronic diseases, including kidney disease, diabetes, and cardiovascular diseases (4, 5). Several studies identified high SUA as an independent risk factor for T2DM, particularly among the Western population (6, 7); however, epidemiological studies reported conflicting results among the Asian population (8–10). For instance, recent cohort studies in China demonstrated that high SUA was linked to an increased risk of T2DM only in women (8, 11).

Overweight/obesity, dyslipidemia, and hypertension often co-exist with T2DM (12, 13) and are also related to high SUA levels (14). A national health survey showed a significant association between elevated SUA levels and the increased prevalence of abdominal obesity, hypertriglyceridemia, and hyperglycemia in the US population (15). A previous study reported that obesity could significantly mediate the association between hyperuricemia and diabetes risk (16), and body mass index (BMI) and dyslipidemia were significant mediators in the association only in women (11). However, the causal relationship between hyperuricemia and prediabetes or diabetes has yet to be explored. It is still unclear whether the increased prediabetes and diabetes risk due to elevated SUA is *via* multiple mediators like obesity, fatty liver, and dyslipidemia. Therefore, we aim to determine the mediating mechanism of their relationship *via* fatty liver disease, high BMI, and dyslipidemia. Also, our study evaluates both single and possible combinations of the mediators’ effects on prediabetes and diabetes with the weighting-based mediation model approach.

## Methods

### Design and setting

The Fuqing cohort aims to investigate the natural history and risk factors of chronic non-communicable diseases, including cancer, diabetes, and fatty liver, among the Chinese population residing in the Southeast coastal region of China. The present study was based on the baseline data collected from the Fuqing cohort participants, which began on 14 July 2020. Seven thousand and nine individuals aged 35 to 75 years old and residing in the 23 rural villages of Gaoshan town were recruited for the study until 31 June 2021.

### Participants

The current analysis excluded subjects with self-reported diabetes and undergoing-treatment diabetes or hyperuricemia cases, and a detailed description of the selection of study participants is presented in **Supplementary Figure 1**. Finally, the dataset for analysis included 5,336 participants (1,870 men and 3,466 women; median age of 57 years). Each participant was interviewed by trained staff using a structured electronic questionnaire, including socio-demographics, lifestyle and dietary habits, history of selected diseases and medication use, and family history of selected diseases. The interview was tape-recorded. The response rate of the Fuqing cohort was 48% for study participants. The ethical committee of Fujian Medical University approved this study [2017-07] and [2020-58], and all participants provided written informed consent before participation in the study.

### Laboratory testing

Each serum sample was measured on an automatic biochemical analyzer (TBA-120FR, TOSHIBA, Japan) with reagents from DiaSys Co., Ltd (Golzheim, Germany). SUA was measured using an enzymatic colorimetric test with the uricase-peroxidase method, and its concentration was measured in mg/dl (1 mg/dl = 59.48 mmol/L). Serum total cholesterol (TC) and triglycerides (TG) were measured using a chromatographic enzymic method in the analyzer. Low-density lipoprotein cholesterol (LDL-C) and high-density lipoprotein cholesterol (HDL-C) were measured using a homogeneous method. Serum creatinine was measured using a kinetic test.

### Definition of outcome and exposure

Participants whose fasting blood glucose levels ≥ 7 mmol/L and/or glucose level after 2 h of oral glucose tolerance test (OGTT) ≥ 11.1 mmol/L and/or glycated hemoglobin A1c (HbA1c) ≥ 6.5% and/or the use of anti-diabetic drugs were classified as having type 2 diabetes, and non-diabetic individuals whose fasting blood glucose levels ≥ 5.6 mmol/L to < 7 mmol/L, or glucose level after 2 h of OGTT ≥ 7.8 mmol/L to < 11.1 mmol/L, or HbA1c ≥ 5.7 to < 6.5% were classified as prediabetes, according to American Diabetes Association criteria (17). Hyperuricemia was defined as SUA concentration > 7.0 mg/dl (416.4 μmol/L) for men or > 6.0 mg/dl (356.9 μmol/L) for women (18).

### Definition, measurements, and classification of mediators

SUA is related to BMI and diabetes, and obesity is considered a mediator in the association between hyperuricemia and diabetes (16). A recent study showed that high BMI and dyslipidemia significantly mediated the association in women (11). Elevated SUA is significantly associated with hyperlipidemia (19) and a higher percentage of fat accumulation in the liver (20). Atherogenic dyslipidemia likely causes incident diabetes (21), and the fatty liver condition is an independent predictor of T2DM in several studies (22). Therefore, we considered high BMI, dyslipidemia, and fatty liver as mediators through which hyperuricemia could increase prediabetes or diabetes risk.

Height was measured, to the nearest 0.1 cm, without shoes, and weight was measured with an electronic bulk composition meter (580515, TANITA Corporation, Japan), to the nearest 100 g, without shoes and with light clothes. BMI was calculated as weight (in kilograms) divided by height (in meters squared). Dyslipidemia was defined as having either or a combination of serum TC ≥ 6.2 mmol/L, LDL‐C ≥ 4.1 mmol/L, HDL‐C < 1 mmol/L, TG ≥ 2.2 mmol/L, and self‐reported use of lipid‐lowering medication, according to the 2007 Chinese guidelines on the prevention and treatment of dyslipidemia (23). Likewise, non-alcoholic fatty liver disease (NAFLD) was diagnosed by experienced doctors using ultrasound images (ALOKA Prosound α7, Japan) and was divided into normal, mild, and moderate-to-severe.

### Definition and classification of covariates

The trained staff took the participants’ blood pressure measurements from their relaxed right arm, which was supported by a table with an electronic sphygmomanometer (OMRON U30 sphygmomanometer, OMRON Healthcare Co, Japan). Each participant was measured twice, and the average of the two measurements was used in the analysis. If the difference between the two measurements were > 5 mmHg, the third measurement was conducted and calculated as the average of two measurements with similar values. Participants whose average blood pressure levels were ≥ 140/90 mmHg or under anti-hypertensive medication were categorized as having hypertension (24). Smoking was categorized into never, past, and current smokers. Likewise, alcohol drinking was classified into never, past, and current users. Physical activities were measured as a metabolic equivalent (MET) per day. The estimated glomerular filtration rate (eGFR) was calculated using blood creatinine value; the calculation method was based on the chronic disease epidemiology method (25).

### Causal mediation analysis

Causal inference methods for mediation analysis are an extension of the traditional approach. First, in the presence of exposure–mediator interaction, total effect (te) is decomposed into direct and indirect effects (controlled or natural) from a potential counterfactual outcomes framework; it develops estimations of these quantities that are not model specific. Second, causal mediation elucidates the primary assumptions to estimate direct and indirect effects, providing clarity to the no unmeasured confounding assumptions. Under the causal mediation approach, sensitivity analyses can be conducted to examine the robustness of findings to violations of these assumptions.

The controlled direct effect (cde) is the effect derived by the contrast between the counterfactual outcome if the individual were exposed at A = a and the counterfactual outcome if the same individual were exposed at A = a*, with the mediator set to a fixed level M = m. The natural direct effect (nde) is the contrast between the counterfactual outcome if the individual were exposed at A = a and the counterfactual outcome if the same individual were exposed at A = a*, with the mediator assuming whatever value it would have taken at the reference value of the exposure A = a*. The pure natural direct effect (pnde) is in the absence of reference interaction while the total natural direct effect (tnde) is the effect including reference interaction. The natural indirect effect (nide) is intuitively defined as the effect of the mediator in the absence of exposure. This effect is the contrast between the counterfactual outcome if the mediator assumed whatever value it would have taken at a value of the exposure A = a and the counterfactual outcome if the mediator assumed whatever value it would have taken at a reference value of the exposure A = a*. The pure natural indirect (pnie) effect is in the absence of mediator interaction, while the total natural indirect effect (tnie) is the effect including mediator interaction. The total effect not only is equal to the sum of the indirect and direct effects but also includes interaction if it exists. Proportion mediated (pm) is defined as the ratio of the total natural indirect effect to the total effect.

Several causal mediation analysis approaches are implemented, including the regression-based approach, the weighting-based approach, the inverse-odds-ratio-weighting approach, the natural effect model, the marginal structural model, and the g-formula approach. A regression-based method estimates the direct and indirect effects under a parametric assumption. It requires the model for the outcome, and the models for each of the mediators are correctly specified; no model for the exposure is needed in the regression approach (26). In contrast, the weighting approach specifies correctly the model for the outcome and the model for the exposure; no models for the mediators are needed. In the regression approach, the model for the outcome, and the models for each of the mediators are required to be correctly specified, whereas no model for the exposure is needed (26). Although this approach deals with when the outcome is binary rather than continuous, it can be used if the mediators are binary (or if some are binary and some are continuous). This weighting approach can be used for any type of outcome, including non-rare binary outcomes; it can also be used regardless of whether there are exposure–mediator or mediator–mediator interactions (26). However, as with other weighting approaches, it works best when the exposure is binary or discrete with only a few levels. If there is a missingness problem in the outcome dataset, natural effect models can deal with it within the counterfactual framework (27).

Some more causal mediation approaches work in their principle and the assumptions under which effect values are calculated for time-varying variables. For example, the marginal structural model is designed to control for the effect of confounding variables that change over time and are affected by previous treatment (28). The parametric g-formula approach can accommodate both mediation and time-varying exposures, mediators, and confounders (29); thus, it constitutes a general approach to mediation analysis with time-varying exposures and mediators.

### Statistical analyses

Continuous and categorical variables are presented as mean values ± standard deviation and frequencies with percentages, respectively. An independent two-sample *t*-test was used to test differences among participants with and without hyperuricemia for continuous variables. The difference in distribution for categorical variables was tested using *χ* ^2^ test. Multinomial logistic regression was performed to examine the association between hyperuricemia and the risk of prediabetes or diabetes, adjusting for potential confounding covariates, namely, age in years, sex (male/female), BMI, fatty liver (none/mild/moderate-to-severe), hypertension (yes/no), dyslipidemia (yes/no), eGFR, alcohol drinking (current/past/never), smoking (current/past/never), and physical activity metabolic equivalent (MET) per day. We used a weighting-based approach because of several reasons (1): our study mediators were binary, ordinal, and continuous (2); our outcome variable (prediabetes or diabetes) was not a rare disease (3); there were unequal distribution of covariates between those with hyperuricemia and without hyperuricemia.

We estimated cde, pnde, tnde, pnie, tnie, te, and pm; the mathematical formula for calculation has been explained in **Supplementary File 1**. The point estimate of each causal effect was obtained by imputing counterfactuals directly. The standard deviations of bootstrapped results are the standard errors of causal effects, and the percentiles of bootstrapped results get the causal effects’ confidence intervals. A two-tailed *p*-value of 5% was considered statistically significant. We performed mediation analysis with the “CMAverse” R package, and all other statistical analyses were executed in R statistical software using its base packages.

## Results

### Prevalence and general characteristics

The proportions for prediabetes and diabetes were 44.3% and 11.5%, respectively, after excluding the previously diagnosed cases of diabetes and the participants under antidiabetic medication. We observed 48.5% and 12.9% prediabetes and diabetes among hyperuricemic while 42.2% and 10.8% prediabetes and diabetes, respectively, among normouricemic individuals; the difference in the distribution was significant (*χ* ^2^ = 34.3, *p* < 0.001). The baseline characteristics of hyperuricemia status are presented in **Table 1**. Men and older participants had a higher chance of having hyperuricemia. Participants with higher BMI, hypertension, dyslipidemia, fatty liver, and lower eGFR were more likely to be hyperuricemic. Likewise, physical activity, smoking, and alcohol drinking were significantly associated with hyperuricemia. We evaluated correlation among exposure, mediators, and confounders with correlation matrix (**Supplementary Figure 2**) and principal component analysis (**Supplementary Figure 3**).

**Table 1:** Characteristics of participants by hyperuricemia status.

Variable	Category	Participants (%) or mean (SD)	Hyperuricemia		
			No (%)	Yes (%)	*p*-value
Age (years)		56.6 (9.8)	56.3 (9.8)	57.1 (9.9)	<0.001
Sex	Male	1,870 (35.0)	1,071 (30.1)	799 (44.9)	<0.001
	Female	3,466 (65.0)	2,484 (69.9)	982 (55.1)	
BMI (kg/m^2^)		24.0 (3.2)	23.5 (3.0)	24.9 (3.3)	<0.001
Hypertension	No	2,931 (55.1)	2,061 (58.1)	870 (49.0)	<0.001
	Yes	2,388 (44.9)	1,484 (41.9)	904 (51.0)	
Dyslipidemia	No	3,557 (66.7)	2,512 (70.7)	1,045 (58.7)	<0.001
	Yes	1,779 (33.3)	1,043 (29.3)	736 (41.3)	
Fatty liver	No	3,610 (68.6)	2,651 (75.4)	959 (54.9)	<0.001
	Mild	1,213 (23.0)	674 (19.1)	539 (30.8)	
	Moderate-to-severe	441 (8.4)	192 (5.5)	249 (14.3)	
eGFR (ml/min/1.73 m^2^)		96.5 (11.7)	98.1 (10.7)	93.4 (12.9)	<0.001
Physical activity (MET/day)		14.0 (13.0)	14.0 (13.1)	14.0 (12.6)	<0.001
Smoking	Never	3,935 (73.8)	2,742 (77.2)	1,193 (67.0)	<0.001
	Ex-smoker	467 (8.8)	263 (7.4)	204 (11.5)	
	Daily	928 (17.4)	546 (15.4)	382 (21.5)	
Alcohol drinking	Never	4,754 (89.2)	3,224 (90.8)	1,530 (85.9)	<0.001
	Former	170 (3.2)	102 (2.9)	68 (3.8)	
	Current	408 (7.6)	225 (6.3)	183 (10.3)	

SD, standard deviation; BMI, body mass index; eGFR, estimated glomerular filtration rate; MET, metabolic equivalent per day; kg, kilogram; m^2^, meter squared; ml, milliliter; min, minute.

### Multinomial analysis

We constructed two multinomial regression models: model 1 adjusted for covariates only (age, sex, hypertension, eGFR, alcohol drinking, smoking, and physical activity), and model 2 additionally adjusted for mediators (BMI, fatty liver, and dyslipidemia). In model 1, SUA level was significantly associated with increased risks for both prediabetes and diabetes. The estimates were attenuated mostly for diabetes in model 2 after further adjusting for mediators; the significant results were constrained to overall and women only. Likewise, hyperuricemic individuals had significantly increased risks of prediabetes or diabetes overall in model 1, while the estimates were attenuated in model 2 and only significant for prediabetes. Stratified analyses by sex showed similar patterns, but significant findings were mostly observed among women. With SUA quintile categories, significant associations were observed for prediabetes and diabetes in model 1, which became attenuated mainly in model 2, especially for diabetes. Stratified analyses by sex showed similar patterns, and again, significant findings were mostly found among women **(** **Table 2** **)**. Furthermore, we stratified the analysis by age into middle-aged adults (<55 years) and older adults (55 years and over), and significant prediabetes risk was observed for the fifth quintile of SUA compared to the first quintile in the overall population (**Supplementary Table 1**).

**Table 2:** Multinomial logistic analysis for prediabetes and diabetes risk in association with serum uric acid.

		Model 1			Model 2	
		Prediabetes OR (95% CI)		Diabetes OR (95% CI)	Prediabetes OR (95% CI)	Diabetes OR (95% CI)
Uric acid (continuous)						
Overall		1.003 (1.002, 1.004)***		1.004 (1.003, 1.005)***	1.002 (1.001, 1.003)***	1.001 (1.000, 1.003)*
Men		1.002 (1.001, 1.003)***		1.003 (1.001, 1.004)**	1.001 (1.000, 1.002)*	1.001 (0.999, 1.003)
Women		1.004 (1.003, 1.005)***		1.005 (1.004, 1.007)***	1.002 (1.001, 1.003)***	1.002 (1.000, 1.004)*
Hyperuricemia (yes *vs*. no)						
Overall		1.52 (1.33, 1.73)***		1.65 (1.35, 2.02)***	1.25 (1.09, 1.43)***	1.13 (0.91, 1.40)
Men		1.38 (1.12, 1.70)***		1.32 (0.94, 1.85)	1.19 (0.96, 1.48)	1.03 (0.73, 1.47)
Women		1.57 (1.32, 1.87)***		1.81 (1.40, 2.34)***	1.29 (1.07, 1.54)**	1.18 (0.90, 1.56)
Uric acid (higher quartile *vs*. the lowest quartile)						
Overall	Q2	1.34 (1.11, 1.62)***		1.44 (1.06, 1.96)*	1.24 (1.02, 1.50)*	1.26 (0.91, 1.73)
	Q3	1.37 (1.13, 1.66)***		1.67 (1.22, 2.27)***	1.16 (0.95, 1.41)	1.21 (0.88, 1.67)
	Q4	1.60 (1.31, 1.96)***		2.00 (1.46, 2.75)***	1.27 (1.03, 1.56)*	1.28 (0.92, 1.78)
	Q5	2.26 (1.82, 2.82)***		2.88 (2.05, 4.05)***	1.58 (1.26, 1.99)***	1.44 (1.00, 2.07)*
*p* for linear trend					<0.001	0.075
Men	Q2	1.19 (0.86, 1.62)		1.29 (0.76, 2.18)	1.10 (0.80, 1.52)	1.15 (0.67, 1.96)
	Q3	1.01 (0.73, 1.38)		1.53 (0.93, 2.54)	0.89 (0.64, 1.24)	1.26 (0.75, 2.12)
	Q4	1.32 (0.96, 1.83)		1.46 (0.86, 2.49)	1.11 (0.80, 1.55)	1.09 (0.63, 1.88)
	Q5	1.82 (1.31, 2.54)***		1.72 (0.99, 2.99)	1.41 (1.00, 1.98)*	1.05 (0.59, 1.87)
*p* for linear trend					0.08	0.837
Women	Q2	1.18 (0.94, 1.50)		1.20 (0.81, 1.77)	1.10 (0.87, 1.40)	1.03 (0.69, 1.53)
	Q3	1.31 (1.03, 1.66)*		1.65 (1.13, 2.41)**	1.14 (0.89, 1.45)	1.28 (0.86, 1.88)
	Q4	1.49 (1.17, 1.89)***		1.74 (1.18, 2.55)***	1.22 (0.95, 1.56)	1.13 (0.75, 1.69)
	Q5	2.09 (1.63, 2.68)***		2.72 (1.86, 3.98)***	1.53 (1.18, 1.98)***	1.40 (0.93, 2.10)
*p* for linear trend					0.001	0.120

OR, odds ratio; CI, confidence interval; Q, quintile; vs, versus.

^*^p ≤ 0.05; ^**^p ≤ 0.01; ^***^p ≤ 0.001.

Model 1 (without adjustment for mediators) adjusted with age, smoking, alcohol drinking, log of physical activity, and in overall group also adjusted with sex.

Model 2 (with adjustment for mediators) further adjusted with fatty liver, body mass index, and dyslipidemia.

### Mediation analysis

**Figure 1:**
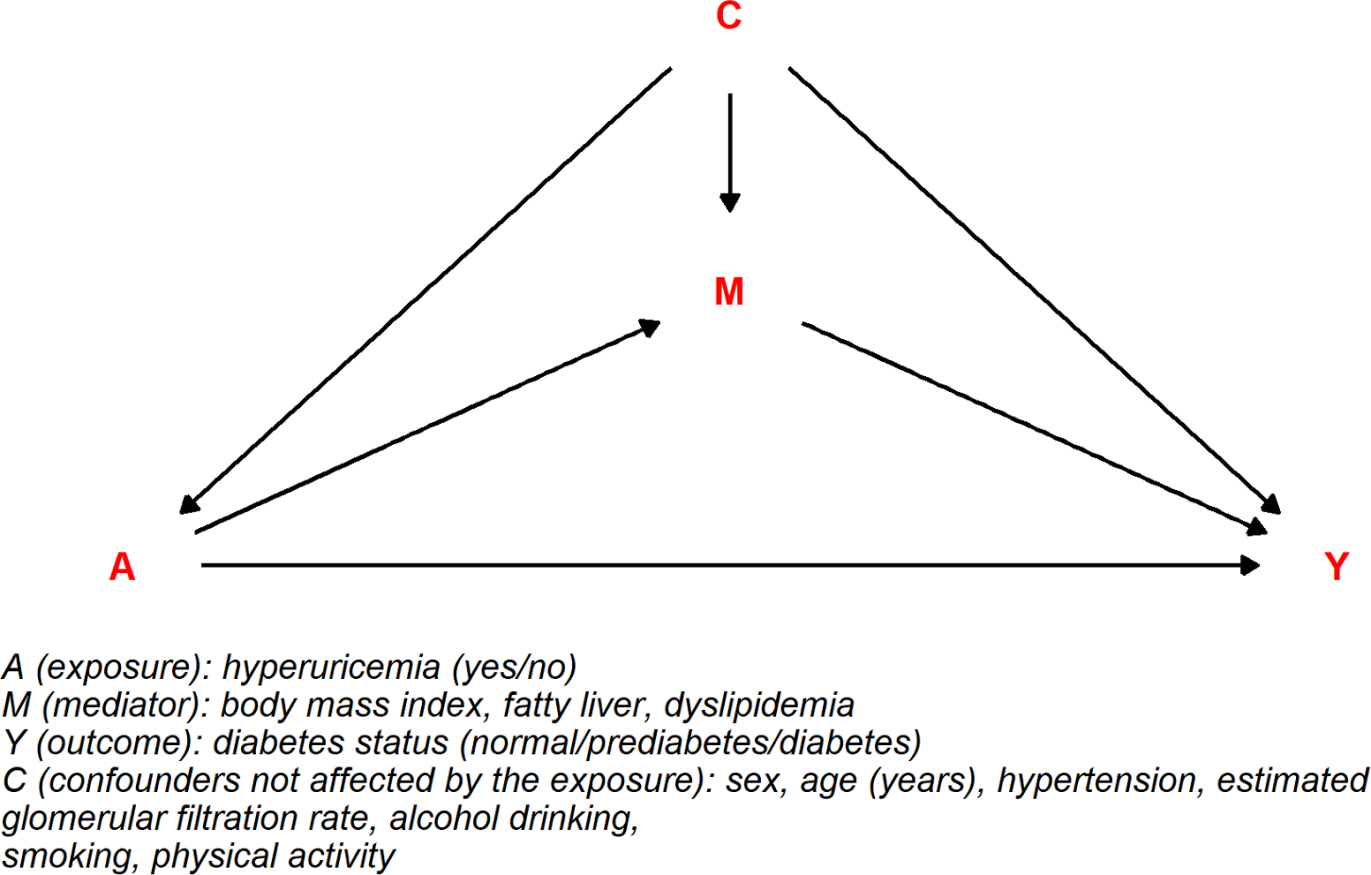
Directed acyclic graph for the combined mediating effect of fatty liver, body mass index, and dyslipidemia on the association between hyperuricemia and diabetes.

A directed acyclic graph was constructed considering fatty liver, BMI, and dyslipidemia as mediators in the association between hyperuricemia and diabetes status (**Figure 1**). All estimates (tnde: 1.11; 95% CI: 1.04, 1.18; tnie: 1.07; 95% CI: 1.05, 1.09; te: 1.18; 95% CI: 1.10, 1.25) were significant for prediabetes risk linked to hyperuricemia while only tnie (1.25; 95% CI: 1.18, 1.33) and te (1.25; 95% CI: 1.05, 1.49) were significant for diabetes risk. The corresponding pm were 42% (95% CI: 27%, 73%) and 100% (95% CI: 57%, 361%) for prediabetes and diabetes, respectively. In sex-wise subgroup analysis, hyperuricemia showed significant tnde only for prediabetes in women and tnie for prediabetes or diabetes risk in both sexes. However, pm was 48% (*p* = 0.008) in men for prediabetes but not significant in men for diabetes while 35% (*p* < 0.001) and 96% (*p* = 0.020) for prediabetes and diabetes in women, respectively **(** **Table 3** **)**. In subgroup analysis among those less than 55 years (middle-aged adults) and equal to or over 55 years (older adults), tnie and pm were significant for prediabetes and diabetes in middle-aged adults. In further age–sex stratification, hyperuricemic middle-aged men and women had statistically significant tnie and pm for prediabetes and diabetes while tnie and pm were significant only in the prediabetes men **(** **Supplementary Table 2** **)**.

**Table 3:** Prediabetes and diabetes causal risk associated with hyperuricemia based on weighted model jointly mediated by dyslipidemia, body mass index, and fatty liver.

Mediators’ parameter	Prediabetes		Diabetes	
	Estimate	95%CI	Estimate	95%CI
Overall				
Controlled direct effect	1.17	(1.07, 1.29)^**^	1.05	(0.87, 1.29)
Pure natural direct effect	1.10	(1.03, 1.18)^**^	1.00	(0.84, 1.19)
Total natural direct effect	1.11	(1.04, 1.18)^***^	0.96	(0.82, 1.14)
Pure natural indirect effect	1.06	(1.04, 1.09)^***^	1.30	(1.23, 1.37)^***^
Total natural indirect effect	1.07	(1.05, 1.09)^***^	1.25	(1.18, 1.33)^***^
Total effect	1.18	(1.10, 1.25)^***^	1.25	(1.05, 1.49)^**^
Proportion mediated (%)	42	(27, 73)^***^	100	(57, 361)^**^
Men				
Controlled direct effect	1.17	(0.95, 1.40)	1.01	(0.71, 1.41)
Pure natural direct effect	1.08	(0.96, 1.20)	0.97	(0.73, 1.26)
Total natural direct effect	1.11	(0.99, 1.22)	0.92	(0.68, 1.23)
Pure natural indirect effect	1.04	(1.01, 1.08)^*^	1.19	(1.10, 1.28)^***^
Total natural indirect effect	1.07	(1.03, 1.10)^***^	1.13	(1.03, 1.24)^**^
Total effect	1.16	(1.03, 1.27)^**^	1.10	(0.82, 1.43)
Proportion mediated (%)	48	(22, 197)^**^	128	(-695, 849)
Women				
Controlled direct effect	1.16	(1.05, 1.29)^**^	1.06	(0.81, 1.32)
Pure natural direct effect	1.11	(1.03, 1.20)^**^	1.01	(0.80, 1.21)
Total natural direct effect	1.11	(1.03, 1.20)^**^	0.99	(0.80, 1.19)
Pure natural indirect effect	1.06	(1.03, 1.08)^***^	1.31	(1.21, 1.42)^***^
Total natural indirect effect	1.05	(1.02, 1.09)^***^	1.28	(1.18, 1.40)^***^
Total effect	1.17	(1.09, 1.27)^***^	1.30	(1.04, 1.56)^*^
Proportion mediated (%)	35	(15, 67)^***^	96	(54, 337)^*^

CI, confidence interval; ^*^P ≤ 0.05; ^**^P ≤ 0.01; ^***^P ≤ 0.001.

Adjusted for age, sex, hypertension, estimated glomerular filtration rate, smoking, alcohol drinking, log of physical activity.

In addition, we evaluated the effect of each mediator and the possible combination of mediators **(** **Supplementary Table 3**). Hyperuricemia showed significant tnde, te, and tnie, including pm, mediated by fatty liver jointly with dyslipidemia (pm = 17%) or BMI (pm = 35%), on prediabetes risk. In contrast, for diabetes risk, the only significant indirect effect was observed mediated by fatty liver disease singly or jointly with either BMI or dyslipidemia, while other mediation parameters were insignificant.

### Effect modification with mediators

Hyperuricemia and SUA quintiles (*p* for linear trend = 0.001) were significantly associated with prediabetes among individuals with mild fatty liver disease compared to those with no fatty liver (*p* for interaction 0.172 for hyperuricemia and 0.073 for SUA quintiles). Hyperuricemia demonstrated a relatively higher prediabetes risk among people with fatty liver and normal blood lipid levels than individuals with no fatty liver and no dyslipidemia (*p* for interaction 0.047). Compared to the lower SUA quintile, the highest SUA quintile showed significant prediabetes risk among people with fatty liver and normal lipid levels (*p* for linear trend 0.017 and interaction 0.010) and fatty liver and dyslipidemia (*p* for linear trend 0.010 and interaction 0.182) than people with no fatty liver and no dyslipidemia. Hyperuricemia showed a higher prediabetes risk among people with fatty liver and non-obesity than people with no fatty liver and non-obesity (*p* for interaction 0.138). Compared to the lowest SUA quintile, the highest SUA quintile demonstrated significant prediabetes risk among people with fatty liver and non-obesity (*p* for linear trend 0.002 and interaction 0.169) and both fatty liver and obesity (*p* for linear trend 0.005 and interaction 0.987) compared to people with no fatty liver and non-obesity. Significant diabetes risk was observed with the highest SUA quintile compared to the lowest among individuals with fatty liver and obesity (*p* for linear trend 0.019 and interaction 0.019) than people with no fatty liver and non-obesity. The above results indicated that increased prediabetes risk was greater among people with fatty liver disease (**Supplementary Tables 4–6**).

### Sensitivity analysis

The mediation estimates were likely influenced by unmeasured confounders, such as environmental exposure to toxicants, dietary factors, and a family history of diabetes; therefore, we performed a sensitivity analysis considering the effect of unmeasured confounders in the association. Overall, we observed high E-values for prediabetes and diabetes risk in men and women, which indicated that only relatively strong unmeasured confounders could change the reported effects (**Supplementary Table 7**).

## Discussion

In a multinomial regression model without considering mediators in the association, we observed that hyperuricemia was significantly associated with a higher risk of prediabetes independent of age, sex, BMI, dyslipidemia, fatty liver, hypertension, eGFR, smoking, alcohol drinking, and physical activity. Furthermore, the association remained significant for prediabetic risk only in women; however, we did not find a significant association between hyperuricemia and increased diabetes risk.

In addition, we investigated the mediation mechanism of association between hyperuricemia and diabetes status: individual and the combined effects of BMI, dyslipidemia, and fatty liver were evaluated. We observed that BMI, dyslipidemia, and fatty liver jointly mediated the association. In the analysis considering these three mediators, hyperuricemia significantly increased the risk directly and indirectly for prediabetes or diabetes; the corresponding pm was 42% (*p* < 0.001) and 100% (*p* = 0.008), respectively. In sex-wise subgroup analysis, these mediators modulated the association significantly for prediabetes or diabetes risk in women (pm = 35% and 96%, respectively), while men had significant pm only for prediabetes (pm = 48%).

An elevated SUA level has been reported with an increased risk for diabetes and prediabetes in the Western population. For instance, cohort studies in the US (6), the Netherlands (7), and Germany (30) showed that hyperuricemia was an independent risk factor for prediabetes or diabetes. A meta-analysis also revealed a higher diabetes risk among hyperuricemic subjects, providing strong evidence that a higher SUA level is independent of other established risk factors for developing T2DM in middle-aged and older people (31).

In contrast, among the Asian population, including the Chinese people, mixed results were revealed (8–11). Using the multinomial logistic regression model, we observed that hyperuricemia was an independent risk factor for prediabetes but not diabetes among adults (aged between 35 and 75 years). The significant risk for prediabetes and not diabetes may be because our analysis excluded diagnosed cases of diabetes and individuals under antidiabetic medication. However, the finding agreed with a study that concludes that serum uric acid is more closely linked to early-phase mechanisms in the development of T2DM than late-phase mechanisms (7). The reported differences in the relative risk among the different gender and populations were probably partly due to the study’s sample size, disease classification, lifestyle, dietary habits, and exposure to environmental conditions that interact with their genetic background. Also, there might be differences in the burden of comorbidities like hypertension, fatty liver, obesity, dyslipidemia, and kidney disease, which directly or indirectly were associated with hyperuricemia and diabetes prevalence.

In subgroup analysis, we demonstrated that hyperuricemic women had a higher chance of having prediabetes only. Recent large cohort studies in the Chinese population reported a higher risk of diabetes in hyperuricemic women but not men (8, 11). Likewise, women with higher uric acid were reported at a higher prediabetes and diabetes risk among the Japanese (9) and Korean populations (10). Elevated SUA levels independently increase prediabetes or diabetes risk among the younger population (6), indicating the potential causal role of SUA at an early age. We also stratified by age group into middle-aged adults (<55 years) and older adults (≥ 55 years) and observed that middle-aged adult women with hyperuricemia were at higher risk of diabetes. The reason might be the different biological pathways, including hormonal differences and the effect of confounders involved in the disease progress (hyperuricemia to diabetes).

By performing weighting-based mediation analysis, we found that higher BMI, fatty liver, and dyslipidemia jointly mediated the association between hyperuricemia and diabetes status. Although the indirect effects remained significant in men and women, the proportion mediated was significant for prediabetes and diabetes in women while only for prediabetes in men. Thus, the finding suggested that the effect of mediators was prominent in women with diabetes risk. In the further stratified analysis of the middle-aged and elderly population, we found that middle-aged women with hyperuricemia were more likely to have prediabetes or diabetes than their counterparts. In our study, excess diabetes risk in middle-aged women (<55 years) might be due to a larger proportion of women during the menopausal stage who might suffer from hormonal changes leading to higher uric acid levels (32). A previous study also revealed a significant correlation between SUA levels and metabolic syndrome, and the association was significant in premenopausal women compared to postmenopausal ones (33).

In addition, we evaluated the effect of individual mediators and the possible combination of mediators. When a single mediator was considered, the mediation effect was too small and insignificant. In opposition to our finding, Han et al. reported that BMI as a single mediator significantly mediated the association between hyperuricemia and diabetes; the mediation proportion was 20% (16). A recent cohort study demonstrated that high BMI and dyslipidemia partially mediated the association in Chinese adult women (11). However, both studies reported their findings analyzing diabetes as a binary variable. A possible combination of two mediators, especially fatty liver with either BMI or dyslipidemia, significantly increased the effect of hyperuricemia on prediabetes risk, indicating that fatty liver condition has a crucial mediating role in the association. The previous study claimed a potentially causal impact of NAFLD on diabetes (34), which supported the idea that fatty liver was a primary mediator in the association.

The previous clinical and experimental studies have shown that higher uric acid mediates vascular changes leading to renal ischemia and renin–angiotensin system stimulation, promoting hypertension, hypertriglyceridemia, and hepatic steatosis through pro-oxidative mechanisms and ultimately the development of insulin resistance and decreased release of insulin leading to T2DM (35), which supports our proposed multiple mediation mechanism. Higher uric acid also augments reactive oxygen species production leading to the loss of transcription factors needed for insulin gene expression, eventually decreasing insulin production and secretion (36).

The present study has several limitations. First, we used cross-sectional data for the cause–effect analysis, which has several inherent study design drawbacks. Second, the traditional non-instrumental variable method for mediation analysis has its methodological problem, including bias due to confounding between exposure, mediator, and outcome. Simplifying some mediators like fatty liver and dyslipidemia into categorical variables introduced measurement error, which biases the indirect effect and thus mediated proportion towards the null. Therefore, the actual mediated proportion of the association between hyperuricemia and prediabetes or diabetes mediated by biological fatty liver and dyslipidemia might be higher than that reported in our study. Furthermore, we analyzed the effect of multiple mediators in the association between hyperuricemia and diabetes status without considering the time effect and the confounder affected by exposures (hyperuricemia).

The previous research showed that persistent hyperuricemia at baseline to follow-up could better predict diabetes risk and cross-lag analysis shows the reverse relation of diabetes to the SUA level (8). Therefore, we analyzed the data excluding self-reported diabetes and individual undergoing-treatment for diabetes and hyperuricemia, possibly removing the effect of reverse causality. We used weighting-based mediation analysis to better predict the causal estimation in the scenario where some mediators like fatty liver and dyslipidemia were simplified into categorical variables. Considering that the estimates were likely to be influenced by unmeasured confounders like family history of diabetes, environmental exposure to toxicants, and dietary factors, we performed a sensitivity analysis that showed relatively large E-values, indicating that considerable unmeasured confounding would be needed to explain away an effect estimate.

Hyperuricemia is associated with higher prediabetes and diabetes risk among the Chinese population, partially mediated by higher BMI, dyslipidemia, and fatty liver. Increased diabetes and prediabetes risks were more prominent in women and middle-aged adults. Among the mediators considered, fatty liver jointly with either dyslipidemia or higher BMI had a robust mediating effect in the association. The findings suggest that further randomized controlled trials are needed to consider treatment options for asymptomatic hyperuricemia, with higher BMI, dyslipidemia, and fatty liver to prevent prediabetes and diabetes risk. Finally, the clinician should be cautious of hyperuricemic patients with higher BMI, dyslipidemia, and fatty liver to avoid the future risk of developing diabetes.

## Data availability statement

The original contributions presented in the study are included in the article/**Supplementary Material**. Further inquiries can be directed to the corresponding author.

## Ethics statement

The ethical committee of Fujian Medical University approved this study [2017-07] and [2020-58]), and all participants provided written informed consent before participation in the study. The patients/participants provided their written informed consent to participate in this study.

## Author contributions

TB and WY contributed to the conception and design of the work. TB, RF, SD, JG and JMG contributed to the task’s acquisition, analysis, or interpretation of data. TB drafted the manuscript. TB, RF, SD, JG, and WY critically revised the manuscript. All authors contributed to the article and approved the submitted version.

## Supplementary material

Supplementary Figure 1 Flowchart diagram of selection of study subjects.

Supplementary Figure 2 Correlation matrix for exposure, mediators, confounders, mediators, and outcome variables under study.

Supplementary Figure 3 Principal component analysis of the study variables (exposure, confounders, mediators, and outcome).
