# Supplementary material for: Fatty liver mediates the association of hyperuricemia with prediabetes and diabetes: a weighting-based mediation analysis
Source: Front Endocrinol (Lausanne). 2023 Apr 12;14:1133515. doi: 10.3389/fendo.2023.1133515 (PMC10130409; doi:10.3389/fendo.2023.1133515)
Supplement: Supplementary file 1 [file DataSheet_1.docx]

**Supplemental file 1. The weighting-based approach and causal effects calculation formulas**

With the weighting-based approach, causal effects were estimated through direct counterfactual imputation estimation by the following steps:

1. Fit a regression model for the distribution of E (Y|A, M, C).
2. If C is not empty, fit a regression model for P(A|C) and obtain P (A = Ai|C = Ci) for i=1,….,n.
3. For i=1,....,n, obtain E [Yi|A = a∗, M = m, C = Ci], E[Yi|A = a, M = m, C = Ci], E[Yi|A = a∗, M = Mi, C = Ci] and E[Yi|A = a, M = Mi, C = Ci] from the regression model in step 1.
4. Impute the counterfactuals E [Ya∗m], E[Yam], E[Ya∗Ma∗], E[YaMa], E[YaMa∗], and E[Ya∗Ma].

- Impute E[Ya∗m] by taking an average of {E [Yi|A = a∗, M = m, C = Ci]} i=1,.....,n.
- Impute E[Yam] by taking an average of {E[Yi|A = a, M = m, C = Ci]}i=1,…..,n.
- Impute E[Ya∗Ma∗] by taking a weighted average of {Yi}i∈{i:Ai=a∗}, and each subject i is given a weight P(A = Ai)/P(A=Ai|C=Ci).
- Impute E[YaMa] by taking a weighted average of {Yi}i∈{i:Ai=a}, and each subject i is given weight P(A=Ai)/P(A=Ai|C=Ci).
- Impute E[YaMa∗] by taking a weighted average of {E[Yi|A =a, M=Mi, C=Ci]}i∈{i:Ai=a∗}, and each subject i is given a weight P(A=Ai)/P(A=Ai|C=Ci).
- Impute E[Ya∗Ma] by taking a weighted average of {E [Yi|A = a∗ M = Mi, C = Ci]}i∈{i:Ai=a}, and each subject i is given a weight P(A=Ai)/P(A=Ai|C=Ci).

1. Calculate causal effects with formulas in the table below.

Table: Causal effect on the ratio scale

| Full name | Abbreviation | Formula |
| --- | --- | --- |
| Controlled Direct Effect | CDE | E[Yam]/[Ya∗m] |
| Pure Natural Direct Effect | PNDE | E[YaM∗a]/E[Ya∗M∗a] |
| Total Natural Direct Effect | TNDE | E[YaMa]/E[Ya∗Ma∗] |
| Pure Natural Indirect Effect | PNIE | E[Ya∗Ma]/E[Ya∗M∗a] |
| Total Natural Indirect Effect | TNIE | E[YaMa]/E[Ya∗M∗a] |
| Total Effect | TE | PNDE x TNIE or TNDE x PNIE |
| Proportion Mediated | PM | PNDE x (TNIE -1))/ (TE-1) |

a and a∗ are the active and control values for A respectively. m is the value at which M is controlled. Ma denotes the counterfactual value of M that would have been observed had A been set to be a. Yam denotes the counterfactual value of Y that would have been observed had A been set to be a, and M to be m. YaMa∗ denotes the counterfactual value of Y that would have been observed had A been set to be a, and M to be the counterfactual value Ma∗. If Y is categorical, E[Y] represents the probability of Y=y where y is a pre-specified value of Y.
