# Supplementary material for: Fatty liver mediates the association of hyperuricemia with prediabetes and diabetes: a weighting-based mediation analysis
Source: Front Endocrinol (Lausanne). 2023 Apr 12;14:1133515. doi: 10.3389/fendo.2023.1133515 (PMC10130409; doi:10.3389/fendo.2023.1133515)
Supplement: Supplementary file 2 [file Table_1.docx]

**Supplementary Table 1. Multinomial logistic analysis on prediabetes and diabetes risk associated with serum uric acid stratified by age**

|  |  | **Young adults (< 55 years)** | |  | **Older adults (≥ 55 years)** | |
| --- | --- | --- | --- | --- | --- | --- |
|  |  | **Prediabetes** | **Diabetes** |  | **Prediabetes** | **Diabetes** |
|  |  | **OR (95% CI)** | **OR (95%CI)** |  | **OR (95%CI)** | **OR (95%CI)** |
| **Uric acid (continuous)** | | |  |  |  |  |
| **Overall** |  | 1.001 (1.000, 1.003) * | 1.001 (0.999, 1.004) |  | 1.001 (1.000, 1.002) | 1.000 (0.999, 1.002) |
| **Men** |  | 1.001 (0.998, 1.003) | 1.000 (0.996, 1.004) |  | 1.001 (0.999, 1.002) | 1.000 (0.998, 1.002) |
| **Women** |  | 1.002 (1.000, 1.004) * | 1.002 (0.999, 1.005) |  | 1.002 (1.000, 1.003) * | 1.001 (0.999, 1.003) |
| **Hyperuricemia (yes *vs.* no)** | | |  |  |  |  |
| **Overall** |  | 1.21 (0.98, 1.50) | 1.39 (0.93, 2.08) |  | 1.13 (0.94, 1.35) | 0.92 (0.71, 1.19) |
| **Men** |  | 1.23 (0.85, 1.77) | 0.75 (0.36, 1.57) |  | 1.02 (0.78, 1.33) | 0.95 (0.63, 1.41) |
| **Women** |  | 1.22 (0.93, 1.59) | 1.76 (1.09, 2.84) * |  | 1.22 (0.95, 1.57) | 0.85 (0.60, 1.21) |
| **Uric acid (higher quintile *vs*. the lowest quintile)** | | | |  |  |  |
| **Overall** | Q2 | 1.31 (0.98, 1.75) | 1.91 (0.99, 3.71) |  | 1.24 (0.97, 1.60) | 0.98 (0.68, 1.41) |
|  | Q3 | 1.13 (0.84, 1.53) | 1.41 (0.71, 2.80) |  | 1.30 (1.00, 1.68) | 0.91 (0.63, 1.32) |
|  | Q4 | 1.17 (0.85, 1.61) | 2.33 (1.19, 4.56) * |  | 1.27 (0.97, 1.66) | 0.97 (0.66, 1.42) |
|  | Q5 | 1.65 (1.15, 2.38) ** | 1.82 (0.84, 3.92) |  | 1.27 (0.95, 1.69) | 0.96 (0.64, 1.44) |
| **P for linear trend** |  | 0.04 | 0.121 |  | 0.170 | 0.889 |
| **Men** | Q2 | 0.93 (0.52, 1.64) | 0.83 (0.27, 2.57) |  | 1.22 (0.83, 1.80) | 1.09 (0.58, 2.04) |
|  | Q3 | 0.98 (0.55, 1.75) | 1.10 (0.37, 3.23) |  | 0.87 (0.59, 1.29) | 1.08 (0.59, 1.98) |
|  | Q4 | 1.23 (0.79, 2.43) | 0.57 (0.18, 1.87) |  | 0.85 (0.57, 1.27) | 1.03 (0.56, 1.90) |
|  | Q5 | 1.13 (0.63, 2.02) | 0.71 (0.23, 2.20) |  | 1.26 (0.83, 1.91) | 1.00 (0.52, 1.92) |
| **P for linear trend** |  | 0.332 | 0.421 |  | 0.843 | 0.904 |
| **Women** | Q2 | 1.10 (0.78, 1.55) | 1.13 (0.50, 2.53) |  | 0.93 (0.67, 1.28) | 0.80 (0.50, 1.29) |
|  | Q3 | 1.32 (0.93, 1.87) | 1.96 (0.93, 4.13) |  | 1.29 (0.92, 1.81) | 1.29 (0.81, 2.05) |
|  | Q4 | 1.06 (0.74, 1.51) | 1.09 (0.49, 2.42) |  | 1.11 (0.79, 1.56) | 0.90 (0.51, 1.45) |
|  | Q5 | 1.38 (0.95, 2.00) | 2.00 (0.93, 4.32) |  | 1.41 (0.98, 2.02) | 1.16 (0.71, 1.89) |
| **P for linear trend** |  | 0.155 | 0.120 |  | 0.048 | 0.526 |

OR: odds ratio; CI: confidence interval; Q: quintile; vs: versus.

*p ≤ 0.05; **p ≤ 0.01; ***p ≤ 0.001.

Adjusted for hypertension, estimated glomerular filtration rate, fatty liver, body mass index, dyslipidemia, smoking, alcohol drinking, log of physical activity, and in overall group also adjusted with sex

**Supplementary Table 2. Prediabetes and diabetes causal risk associated with hyperuricemia based on a weighted model jointly mediated by body mass index, dyslipidemia, and fatty liver, stratified by age**

|  | **< 55 years** | | |  | **≥ 55 years** | | |
| --- | --- | --- | --- | --- | --- | --- | --- |
| **Overall** | **Prediabetes**  **Estimate (95%CI)** |  | **Diabetes**  **Estimate (95%CI)** |  | **Prediabetes**  **Estimate (95%CI)** |  | **Diabetes**  **Estimate (95%CI)** |
|  |  |  |  |  |  |  |  |
| Controlled direct effect | 1.23 (1.02, 1.36) * |  | 1.44 (0.99, 2.16) |  | 1.11 (0.98, 1.27) |  | 0.93 (0.76, 1.15) |
| Pure natural direct effect | 1.14 (0.99, 1.28) |  | 1.29 (0.94, 1.89) |  | 1.07 (0.99, 1.16) |  | 0.92 (0.76, 1.11) |
| Total natural direct effect | 1.13 (0.99, 1.27) |  | 1.33 (0.95, 1.92) |  | 1.08 (1.00, 1.17) * |  | 0.87 (0.73, 1.05) |
| Pure natural indirect effect | 1.13 (1.07, 1.18) *** |  | 1.34 (1.17, 1.57) *** |  | 1.02 (1.00, 1.05) * |  | 1.24 (1.16, 1.32) *** |
| Total natural indirect effect | 1.12 (1.05, 1.17) *** |  | 1.38 (1.19, 1.62) *** |  | 1.03 (1.01, 1.06) *** |  | 1.17 (1.10, 1.27) *** |
| Total effect | 1.23 (1.10, 1.39) *** |  | 1.78 (1.27, 2.60) *** |  | 1.11 (1.03, 1.2) ** |  | 1.08 (0.91, 1.29) |
| Proportion mediated (%) | 50 (25, 106) *** |  | 63 (36, 121) *** |  | 33 (10, 101) ** |  | 207 (-1922, 1968) |
| **Men** |  |  |  |  |  |  |  |
| Controlled direct effect | 1.4 (0.97, 1.98) |  | 0.95 (0.46, 2.03) |  | 1.07 (0.86, 1.36) |  | 1.01 (0.69, 1.38) |
| Pure natural direct effect | 1.17 (0.92, 1.47) |  | 0.85 (0.44, 1.6) |  | 1.04 (0.92, 1.19) |  | 1.02 (0.73, 1.38) |
| Total natural direct effect | 1.27 (1.01, 1.58) * |  | 0.84 (0.43, 1.54) |  | 1.05 (0.93, 1.19) |  | 0.95 (0.67, 1.27) |
| Pure natural indirect effect | 1.05 (0.95, 1.15) |  | 1.20 (0.93, 1.52) |  | 1.04 (1.01, 1.08) ** |  | 1.17 (1.08, 1.29) *** |
| Total natural indirect effect | 1.13 (1.06, 1.23) *** |  | 1.18 (0.92, 1.52) |  | 1.06 (1.01, 1.10) ** |  | 1.09 (0.99, 1.20) |
| Total effect | 1.33 (1.05, 1.65) * |  | 0.99 (0.54, 1.86) |  | 1.1 (0.97, 1.24) |  | 1.11 (0.8, 1.48) |
| Proportion mediated (%) | 48 (21, 194) * |  | -65 (-721, 1054) |  | 61 (-244, 536) |  | 82 (-882, 482) |
| **Women** |  |  |  |  |  |  |  |
| Controlled direct effect | 1.13 (0.91, 1.33) |  | 1.61 (1.06, 2.38) * |  | 1.16 (0.98, 1.38) |  | 0.89 (0.67, 1.19) |
| Pure natural direct effect | 1.11 (0.95, 1.3) |  | 1.56 (1.04, 2.27) * |  | 1.11 (1.01, 1.22) * |  | 0.84 (0.66, 1.06) |
| Total natural direct effect | 1.1 (0.96, 1.27) |  | 1.45 (0.98, 2.1) |  | 1.12 (1.01, 1.22) * |  | 0.86 (0.67, 1.09) |
| Pure natural indirect effect | 1.12 (1.07, 1.19) *** |  | 1.49 (1.25, 1.79) *** |  | 1.02 (0.99, 1.05) |  | 1.27 (1.17, 1.38) *** |
| Total natural indirect effect | 1.11 (1.05, 1.19) *** |  | 1.39 (1.17, 1.67) *** |  | 1.02 (0.99, 1.05) |  | 1.23 (1.12, 1.35) *** |
| Total effect | 1.24 (1.08, 1.42) ** |  | 2.17 (1.39, 3.28) *** |  | 1.13 (1.04, 1.25) ** |  | 1.05 (0.84, 1.34) |
| Proportion mediated (%) | 51 (21, 150) *** |  | 52 (28, 92) *** |  | 16 (-6, 84) |  | 375 (-1452, 2032) |

CI: confidence interval

*p ≤ 0.05; **p ≤ 0.01; ***p ≤ 0.001.

Adjusted with age, hypertension, estimated glomerular filtration rate, smoking, alcohol drinking, log of physical activity, and in the overall also adjusted with sex

**Supplementary Table 3. Prediabetes and diabetes causal risk associated with hyperuricemia singly and jointly mediated by dyslipidemia, body mass index, fatty liver**

|  | **Prediabetes** | | | **Diabetes** | | |
| --- | --- | --- | --- | --- | --- | --- |
| **Mediator’s parameter** | **Estimate** | **95%CI** | **P-value** | **Estimate** | **95%CI** | **P-value** |
| **BMI*** |  |  |  |  |  |  |
| Controlled direct effect | 1.15 | 1.05, 1.26 | **0.004** | 1.03 | 0.87, 1.22 | 0.704 |
| Pure natural direct effect | 1.10 | 1.03, 1.17 | **0.008** | 1.00 | 0.86, 1.18 | 0.848 |
| Total natural direct effect | 1.10 | 1.03, 1.18 | **0.008** | 0.98 | 0.84, 1.16 | 0.868 |
| Pure natural indirect effect | 1.01 | 1.00, 1.03 | **0.028** | 1.03 | 0.99, 1.06 | 0.092 |
| Total natural indirect effect | 1.01 | 0.99, 1.04 | 0.204 | 1.00 | 0.95, 1.05 | 0.980 |
| Total effect | 1.12 | 1.04, 1.19 | **0.004** | 1.01 | 0.86, 1.19 | 0.872 |
| Proportion mediated (%) | 13 | -11, 46 | 0.208 | 8 | -350, 557 | 0.916 |
| **Dyslipidemia**** |  |  |  |  |  |  |
| Controlled direct effect | 1.11 | 1.04, 1.18 | **0.004** | 0.99 | 0.84, 1.18 | 0.924 |
| Pure natural direct effect | 1.10 | 1.03, 1.17 | **0.004** | 1.01 | 0.87, 1.19 | 0.900 |
| Total natural direct effect | 1.10 | 1.04, 1.18 | **0.008** | 0.97 | 0.82, 1.16 | 0.800 |
| Pure natural indirect effect | 1.00 | 0.99, 1.02 | 0.704 | 1.04 | 1.01, 1.07 | **0.008** |
| Total natural indirect effect | 1.00 | 0.98, 1.03 | 0.784 | 1.00 | 0.95, 1.07 | 0.864 |
| Total effect | 1.10 | 1.04, 1.18 | **<0.001** | 1.01 | 0.85, 1.21 | 0.864 |
| Proportion mediated (%) | 5 | -32, 30 | 0.784 | 44 | -329, 284 | 0.768 |
| **Fatty liver***** |  |  |  |  |  |  |
| Controlled direct effect | 1.11 | 1.03, 1.19 | **0.004** | 0.99 | 0.84, 1.17 | 0.948 |
| Pure natural direct effect | 1.11 | 1.03, 1.18 | **0.008** | 1.00 | 0.85, 1.18 | 0.984 |
| Total natural direct effect | 1.11 | 1.03, 1.18 | **0.008** | 0.97 | 0.81, 1.15 | 0.728 |
| Pure natural indirect effect | 1.01 | 1.00, 1.03 | 0.072 | 1.10 | 1.05, 1.15 | **<0.001** |
| Total natural indirect effect | 1.01 | 0.99, 1.03 | 0.268 | 1.06 | 1.00, 1.12 | **0.048** |
| Total effect | 1.12 | 1.04, 1.20 | **0.004** | 1.07 | 0.90, 1.26 | 0.456 |
| Proportion mediated (%) | 11 | -10, 42 | 0.264 | 98 | -881, 925 | 0.456 |
| **BMI and Dyslipidemia****** |  |  |  |  |  |  |
| Controlled direct effect | 1.16 | 1.05, 1.27 | **<0.001** | 1.03 | 0.87, 1.22 | 0.748 |
| Pure natural direct effect | 1.10 | 1.02, 1.17 | **0.012** | 1.01 | 0.85, 1.18 | 0.956 |
| Total natural direct effect | 1.10 | 1.02, 1.17 | **0.004** | 0.97 | 0.82, 1.15 | 0.728 |
| Pure natural indirect effect | 1.02 | 1.00, 1.03 | **0.012** | 1.05 | 1.01, 1.09 | **0.016** |
| Total natural indirect effect | 1.02 | 1.00, 1.04 | 0.068 | 1.02 | 0.96, 1.08 | 0.608 |
| Total effect | 1.12 | 1.04, 1.19 | **0.004** | 1.02 | 0.87, 1.21 | 0.844 |
| Proportion mediated (%) | 17 | -1, 50 | 0.064 | 68 | -607, 281 | 0.812 |
| **Fatty liver and Dyslipidemia******* | |  |  |  |  |  |
| Controlled direct effect | 1.12 | 1.05, 1.19 | **<0.001** | 1.00 | 0.81, 1.18 | 0.948 |
| Pure natural direct effect | 1.11 | 1.04, 1.18 | **<0.001** | 1.00 | 0.84, 1.19 | 0.960 |
| Total natural direct effect | 1.11 | 1.04, 1.18 | **<0.001** | 0.96 | 0.79, 1.12 | 0.644 |
| Pure natural indirect effect | 1.02 | 1.00, 1.04 | **0.048** | 1.14 | 1.09, 1.20 | **<0.001** |
| Total natural indirect effect | 1.02 | 1.00, 1.05 | **0.044** | 1.09 | 1.03, 1.16 | **<0.001** |
| Total effect | 1.13 | 1.06, 1.21 | **<0.001** | 1.10 | 0.92, 1.30 | 0.308 |
| Proportion mediated (%) | 17 | 0, 45 | **0.044** | 95 | -870, 988 | 0.308 |
| **BMI and Fatty liver******** |  |  |  |  |  |  |
| Controlled direct effect | 1.16 | 1.07, 1.28 | **<0.001** | 1.04 | 0.87, 1.24 | 0.700 |
| Pure natural direct effect | 1.11 | 1.04, 1.18 | **<0.001** | 0.99 | 0.84, 1.16 | 0.900 |
| Total natural direct effect | 1.11 | 1.05, 1.18 | **<0.001** | 0.97 | 0.82, 1.14 | 0.744 |
| Pure natural indirect effect | 1.05 | 1.03, 1.07 | **<0.001** | 1.20 | 1.15, 1.27 | **<0.001** |
| Total natural indirect effect | 1.05 | 1.03, 1.08 | **<0.001** | 1.18 | 1.11, 1.26 | **<0.001** |
| Total effect | 1.16 | 1.11, 1.24 | **<0.001** | 1.17 | 0.99, 1.37 | 0.072 |
| Proportion mediated (%) | 35 | 20, 59 | **<0.001** | 104 | -420, 623 | 0.072 |

CI: confidence interval; bold value indicates significant *P*-value less than 0.05

Adjusted for age, sex, hypertension, estimated glomerular filtration rate, smoking, alcohol drinking, log of physical activity

Further adjusted for *dyslipidemia and fatty liver; ** body mass index and fatty liver; ***dyslipidemia and fatty liver; ****fatty liver; ***** body mass index; ****** dyslipidemia

**Supplementary Table 4. Effect modification of association of serum uric acid with diabetes status by fatty liver**

|  |  | **No fatty liver (n = 3610)** | | **Mild fatty liver (n = 1213)** | | **Moderate-to-severe fatty liver (n = 441)** | |
| --- | --- | --- | --- | --- | --- | --- | --- |
|  |  | **Prediabetes**  **OR (95%CI)** | **Diabetes**  **OR (95%CI)** | **Prediabetes OR (95%CI)** | **Diabetes**  **OR (95%CI)** | **Prediabetes OR (95%CI)** | **Diabetes**  **OR (95%CI)** |
| **Hyperuricemia** |  |  |  |  |  |  |  |
|  | No | Ref | Ref | Ref | Ref | Ref | Ref |
|  | Yes | 1.15 (0.97, 1.36) | 1.11 (0.82, 1.51) | 1.44 (1.08, 1.92) * | 1.17 (0.79, 1.72) | 1.48 (0.87, 2.50) | 1.41 (0.77, 2.57) |
| ***P* for interaction** |  |  |  | 0.172 | 0.927 | 0.258 | 0.470 |
| **Uric acid quintile** |  |  |  |  |  |  |  |
|  | Q1 | Ref | Ref | Ref | Ref | Ref | Ref |
|  | Q2 | 1.08 (0.86, 1.35) | 1.05 (0.68, 1.62) | 1.56 (1.02, 2.40) * | 1.66 (0.94, 2.93) | 1.27 (0.57, 2.86) | 0.85 (0.35, 2.07) |
|  | Q3 | 1.19 (0.94, 1.49) | 1.34 (0.88, 2.04) | 1.82 (1.17, 2.82) ** | 1.87 (1.05, 3.33) * | 1.07 (0.47, 2.41) | 0.79 (0.32, 1.95) |
|  | Q4 | 1.11 (0.87, 1.40) | 1.07 (0.68, 1.68) | 1.86 (1.17, 2.94) ** | 1.64 (0.88, 3.04) | 2.13 (0.87, 5.20) | 1.88 (0.71, 4.97) |
|  | Q5 | 1.22 (0.94, 1.58) | 1.38 (0.86, 2.22) | 2.39 (1.44, 3.96) *** | 1.75 (0.89, 3.44) | 2.01 (0.80, 5.08) | 1.13 (0.39, 3.25) |
| ***P* for linear trend** |  | 0.172 | 0.235 | 0.001 | 0.162 | 0.079 | 0.398 |
| ***P* for interaction** |  |  |  | 0.073 | 0.960 | 0.213 | 0.940 |

OR: Odds ratio; CI: confidence interval

^*^*P* ≤ 0.05; ^**^*P* ≤ 0.01; ^***^*P* ≤ 0.001

Adjusted for age, sex, body mass index, dyslipidemia, hypertension, estimated glomerular filtration rate, smoking, alcohol drinking, log of physical activity

**Supplementary Table 5. Effect modification of association of serum uric acid with diabetes status by fatty liver and dyslipidemia**

|  |  | **No fatty liver and no dyslipidemia (n = 2725)** | | **No fatty liver and dyslipidemia (n = 885)** | | **Fatty liver and no dyslipidemia (n = 791)** | | **Fatty liver and dyslipidemia (n = 863)** | |
| --- | --- | --- | --- | --- | --- | --- | --- | --- | --- |
|  |  | **Prediabetes OR (95%CI)** | **Diabetes**  **OR (95%CI)** | **Prediabetes OR (95%CI)** | **Diabetes**  **OR (95%CI)** | **Prediabetes OR (95%CI)** | **Diabetes**  **OR (95%CI)** | **Prediabetes OR**  **(95%CI)** | **Diabetes**  **OR (95%CI)** |
| **Hyperuricemia** |  |  |  |  |  |  |  |  |  |
|  | No | Ref | Ref | Ref | Ref | Ref | Ref | Ref | Ref |
|  | Yes | 1.09 (0.89, 1.32) | 1.13 (0.77, 1.66) | 1.32 (0.94, 1.86) | 1.08 (0.63, 1.83) | 1.62 (1.14, 2.31) ** | 1.47 (0.91, 2.35) | 1.32 (0.92, 1.89) | 1.10 (0.71, 1.70) |
| ***P* for interaction** | |  |  | 0.827 | 0.837 | 0.047 | 0.379 | 0.318 | 0.572 |
| **Uric acid quintile** |  |  |  |  |  |  |  |  |  |
|  | Q1 | Ref | Ref | Ref | Ref | Ref | Ref | Ref | Ref |
|  | Q2 | 1.07 (0.82, 1.38) | 1.08 (0.64, 1.81) | 1.33 (0.83, 2.12) | 1.23 (0.58, 2.64) | 1.93 (1.14, 3.25) * | 1.61 (0.81, 3.19) | 1.21 (0.69, 2.11) | 1.32 (0.68, 2.55) |
|  | Q3 | 1.15 (0.89, 1.50) | 1.01 (0.59, 1.72) | 1.34 (0.84, 2.16) | 1.87 (0.90, 3.87) | 1.64 (0.97, 2.79) | 1.41 (0.70, 2.83) | 1.09 (0.63, 1.91) | 1.11 (0.57, 2.17) |
|  | Q4 | 1.09 (0.83, 1.44) | 1.08 (0.63, 1.87) | 1.11 (0.68, 1.82) | 1.07 (0.48, 2.38) | 2.40 (1.38, 4.20) *** | 1.45 (0.68, 3.09) | 1.42 (0.79, 2.55) | 1.49 (0.74, 3.01) |
|  | Q5 | 1.09 (0.81, 1.48) | 1.18 (0.66, 2.11) | 1.80 (1.04, 3.10) * | 2.05 (0.89, 4.75) | 2.08 (1.13, 3.82) * | 1.91 (0.87, 4.20) | 1.43 (1.28, 4.62) ** | 1.68 (0.77, 3.68) |
| ***P* for linear trend** | | 0.567 | 0.632 | 0.112 | 0.185 | 0.017 | 0.202 | 0.010 | 0.200 |
| ***P* for interaction** | |  |  | 0.191 | 0.670 | 0.010 | 0.487 | 0.182 | 0.387 |

OR: Odds ratio; CI: confidence interval

^*^*P* ≤ 0.05; ^**^*P* ≤ 0.01; ^***^*P* ≤ 0.001

Adjusted for age, sex, body mass index, hypertension, estimated glomerular filtration rate, smoking, alcohol drinking, log of physical activity

**Supplementary Table 6. Effect modification of association of serum uric acid with diabetes status by fatty liver and obesity**

|  |  | **No fatty liver and no obesity (n = 3433)** | | **No fatty liver and obesity (n = 176)** | | **Fatty liver and no obesity (n = 1143)** | | **Fatty liver and obesity (n = 511)** | |
| --- | --- | --- | --- | --- | --- | --- | --- | --- | --- |
|  |  | **Prediabetes OR (95%CI)** | **Diabetes**  **OR (95%CI)** | **Prediabetes OR (95%CI)** | **Diabetes**  **OR (95%CI)** | **Prediabetes OR (95%CI)** | **Diabetes**  **OR (95%CI)** | **Prediabetes OR (95%CI)** | **Diabetes**  **OR (95%CI)** |
| **Hyperuricemia** |  |  |  |  |  |  |  |  |  |
|  | No | Ref | Ref | Ref | Ref | Ref | Ref | Ref | Ref |
|  | Yes | 1.15 (0.97, 1.37) | 1.17 (0.86, 1.61) | 1.73 (0.82, 3.64) | 0.66 (0.18, 2.46) | 1.58 (1.17, 2.13) ** | 1.28 (0.87, 1.89) | 1.35 (0.85, 2.14) | 1.29 (0.73, 2.25) |
| ***P* for interaction** | |  |  | 0.190 | 0.329 | 0.138 | 0.863 | 0.333 | 0.252 |
| **Uric acid quintile** |  |  |  |  |  |  |  |  |  |
|  | Q1 | Ref | Ref | Ref | Ref | Ref | Ref | Ref | Ref |
|  | Q2 | 1.07 (0.85, 1.35) | 1.11 (0.70, 1.74) | 1.11 (0.37, 3.34) | 1.22 (0.27, 5.42) | 1.22 (0.78, 1.89) | 1.25 (0.72, 2.18) | 1.29 (0.64, 2.60) | 1.64 (0.70, 3.81) |
|  | Q3 | 1.30 (1.02, 1.64) | 1.47 (0.94, 2.28) | 0.68 (0.23, 2.02) | 0.49 (0.08, 2.89) | 1.88 (1.19, 2.97) ** | 1.55 (0.86, 2.78) | 1.13 (0.56, 2.30) | 1.26 (0.53, 3.01) |
|  | Q4 | 1.09 (0.86, 1.39) | 1.14 (0.71, 1.82) | 1.63 (0.53, 5.04) | 0.39 (0.05, 3.29) | 1.69 (1.05, 2.72) * | 1.38 (0.74, 2.55) | 1.65 (0.78, 3.50) | 2.58 (1.06, 6.28) * |
|  | Q5 | 1.25 (0.95, 1.63) | 1.58 (0.97, 2.58) | 1.47 (0.39, 5.53) | 0.35 (0.03, 4.18) | 2.19 (1.31, 3.67) ** | 1.31 (0.66, 2.59) | 3.63 (1.52, 8.70) ** | 3.21 (1.13, 9.13) * |
| ***P* for linear trend** | | 0.139 | 0.097 | 0.451 | 0.271 | 0.002 | 0.430 | 0.005 | 0.019 |
| ***P* for interaction** | |  |  | 0.244 | 0.135 | 0.169 | 0.277 | 0.987 | 0.019 |

OR: Odds ratio; CI: confidence interval;

^*^*P* ≤ 0.05; ^**^*P* ≤ 0.01; ^***^*P* ≤ 0.001

Adjusted for age, sex, dyslipidemia, hypertension, estimated glomerular filtration rate, smoking, alcohol drinking, log of physical activity

**Supplementary Table 7. Sensitivity analysis for the effects of unmeasured confounding variables on the association between hyperuricemia and diabetes conditions**

|  | | **Controlled direct effect** | **Pure natural direct effect** | **Total natural direct effect** | **Pure natural indirect effect** | **Total natural indirect effect** | **Total effect** |
| --- | --- | --- | --- | --- | --- | --- | --- |
| Overall | |  |  |  |  |  |  |
|  | Prediabetes estimate (95%CI) | 1.17 (1.07, 1.29) ^**^ | 1.10 (1.03, 1.18) ^**^ | 1.11 (1.04, 1.18) ^***^ | 1.06 (1.04, 1.08) ^***^ | 1.07 (1.05, 1.09) ^***^ | 1.18 (1.10, 1.25) ^***^ |
|  | Prediabetes E-value estimate | 1.62 | 1.44 | 1.46 | 1.32 | 1.34 | 1.64 |
|  | Diabetes estimate (95%CI) | 1.05 (0.87, 1.29) | 1.00 (0.84, 1.19) | 0.96 (0.82, 1.14) | 1.30 (1.23, 1.37) ^***^ | 1.25 (1.18, 1.33) ^***^ | 1.25 (1.05, 1.49) ^**^ |
|  | Diabetes E-value estimate | 1.28 | 1.05 | 1.24 | 1.92 | 1.81 | 1.80 |
| Men | |  |  |  |  |  |  |
|  | Prediabetes estimate (95%CI) | 1.17 (0.95, 1.40) | 1.08 (0.96, 1.20) | 1.11 (0.99, 1.22) | 1.07 (1.03, 1.10) ^*^ | 1.07 (1.03, 1.10) ^***^ | 1.16 (1.03, 1.27) ^**^ |
|  | Prediabetes E-value estimate | 1.62 | 1.38 | 1.46 | 1.25 | 1.34 | 1.58 |
|  | Diabetes estimate (95%CI) | 1.01 (0.71, 1.41) | 0.97 (0.73, 1.26) | 0.92 (0.68, 1.23) | 1.19 (1.10, 1.28) ^***^ | 1.13 (1.03, 1.24) ^**^ | 1.10 (0.82, 1.43) |
|  | Diabetes E-value estimate | 1.13 | 1.20 | 1.39 | 1.67 | 1.51 | 1.42 |
| Women | |  |  |  |  |  |  |
|  | Prediabetes estimate (95%CI) | 1.16 (1.05, 1.29) ^**^ | 1.11 (1.03, 1.20) ^**^ | 1.11 (1.03, 1.20) ^**^ | 1.06 (1.03, 1.08) ^***^ | 1.05 (1.02, 1.09) ^***^ | 1.17 (1.09, 1.26) ^***^ |
|  | Prediabetes E-value estimate | 1.60 | 1.47 | 1.46 | 1.30 | 1.29 | 1.63 |
|  | Diabetes estimate (95%CI) | 1.06 (0.81, 1.32) | 1.01 (0.80, 1.21) | 0.99 (0.80, 1.19) | 1.31 (1.21, 1.42) ^***^ | 1.28 (1.18, 1.40) ^***^ | 1.30 (1.04, 1.56) ^*^ |
|  | Diabetes E-value estimate | 1.31 | 1.12 | 1.10 | 1.94 | 1.88 | 1.91 |

CI: confidence interval

^*^*P* ≤ 0.05; ^**^*P* ≤ 0.01; ^***^*P* ≤ 0.001

Adjusted for age, sex, hypertension, estimated glomerular filtration rate, smoking, alcohol drinking, log of physical activity
