# Supplementary figures and images for: Fatty liver mediates the association of hyperuricemia with prediabetes and diabetes: a weighting-based mediation analysis
Source: Front Endocrinol (Lausanne). 2023 Apr 12;14:1133515. doi: 10.3389/fendo.2023.1133515 (PMC10130409; doi:10.3389/fendo.2023.1133515)

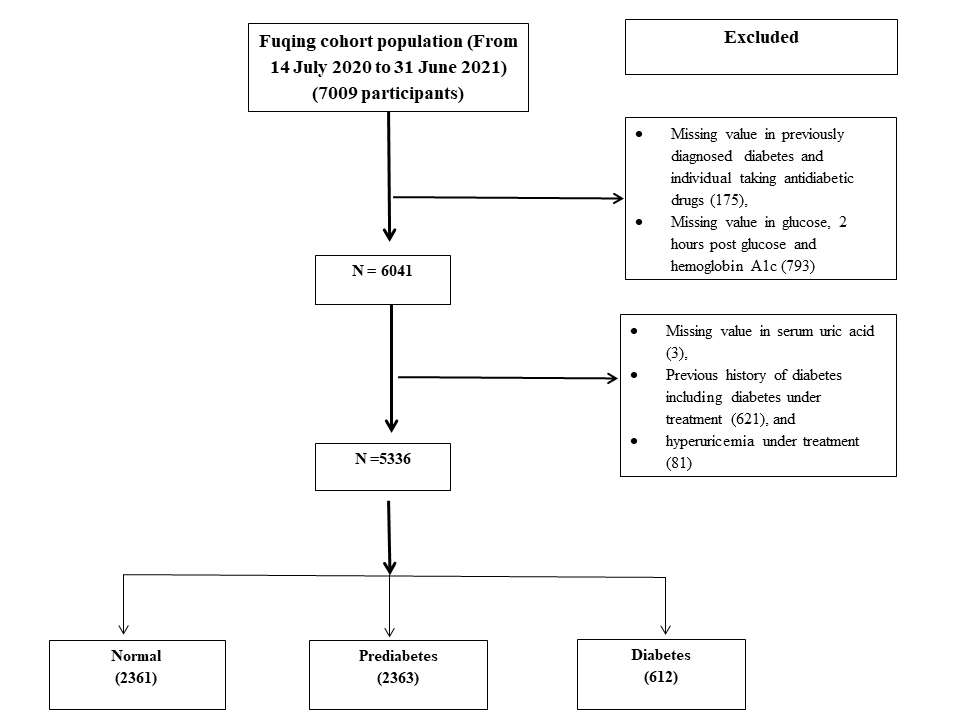

Supplement: Supplementary Figure 1 — Flowchart diagram of selection of study subjects. [file Image_1.tif]

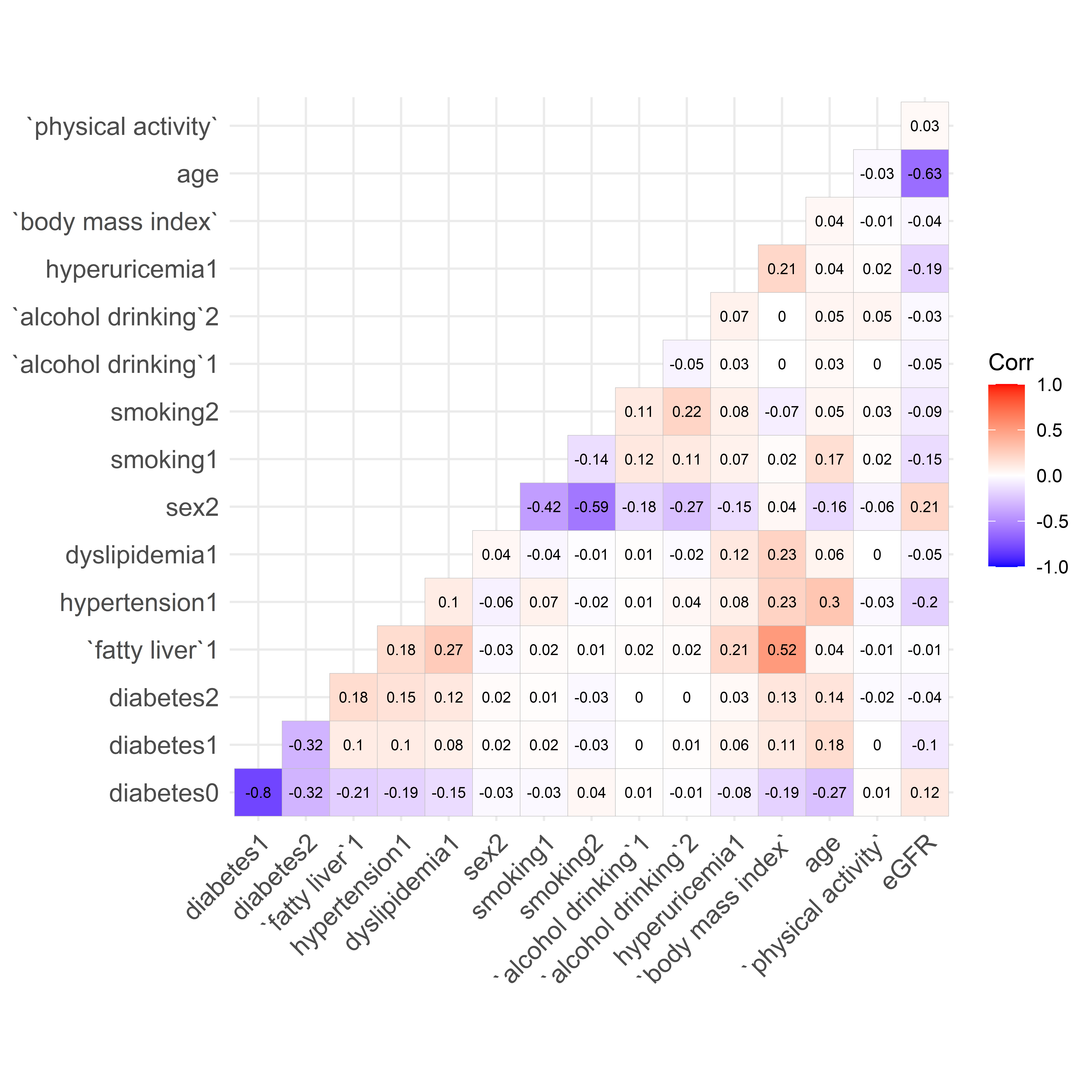

Supplement: Supplementary Figure 2 — Correlation matrix for exposure, mediators, confounders, mediators, and outcome variables under study. [file Image_2.tif]

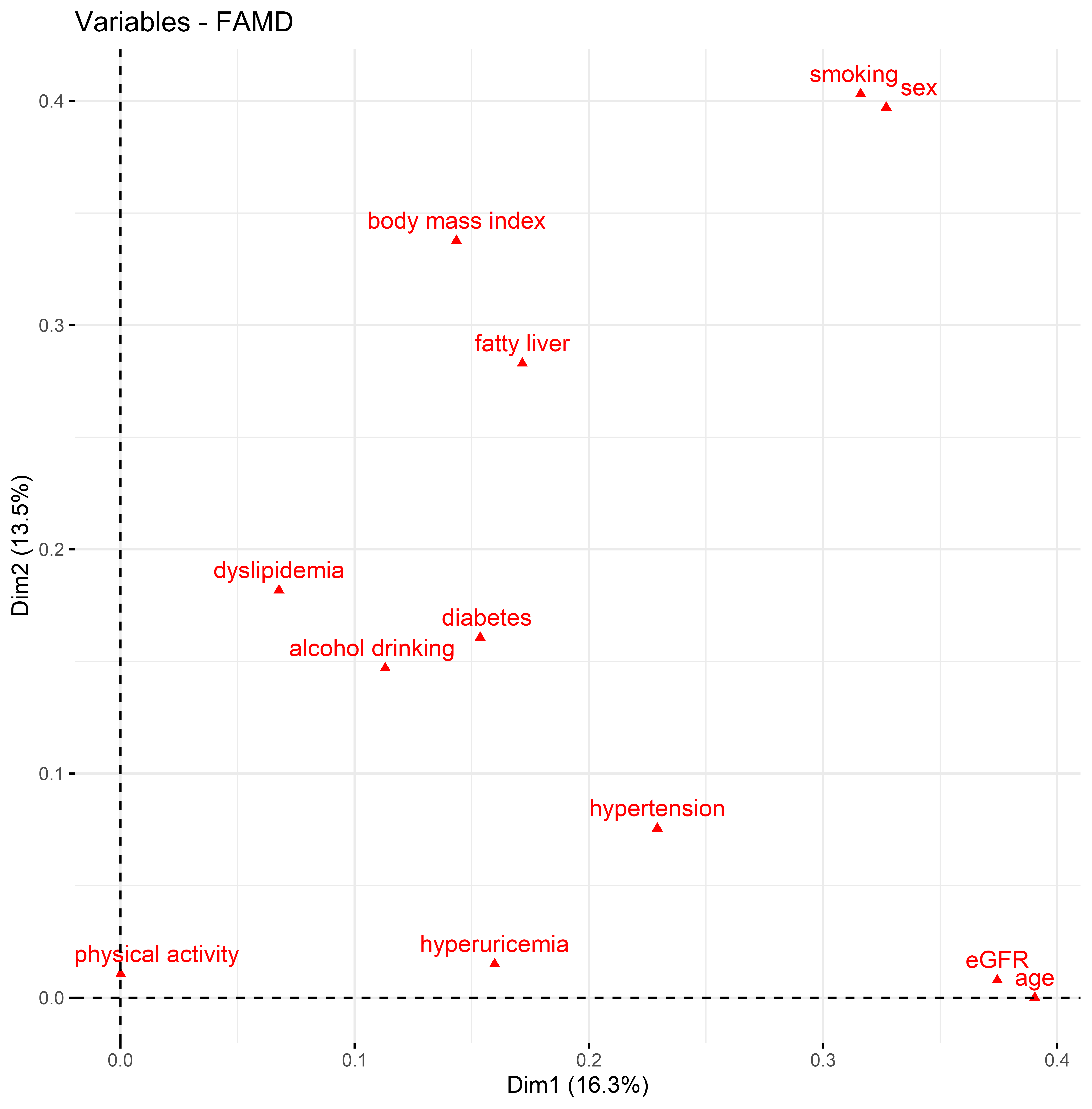

Supplement: Supplementary Figure 3 — Principal component analysis of the study variables (exposure, confounders, mediators, and outcome). [file Image_3.tif]
